# Mechanism of exacerbation of traumatic brain injury under warfarin anticoagulation in male mice

**DOI:** 10.1371/journal.pone.0314765

**Published:** 2024-12-05

**Authors:** Yuki Tatara, Ken-ichiro Nakao, Ryo Shimada, Kazuhiko Kibayashi

**Affiliations:** Department of Forensic Medicine, School of Medicine, Tokyo Women’s Medical University, Tokyo, Japan; Julius-Maximilians-Universitat Wurzburg, GERMANY

## Abstract

**Introduction:**

Traumatic brain injury (TBI) is exacerbated in patients on antithrombotic medications, with warfarin leading to increased bleeding in some cases. However, the extent to which this bleeding increases lethality and its long-term effects remain unclear. This study aimed to investigate the exacerbation of TBI by warfarin treatment and comprehensively evaluate the impact of TBI on the anticoagulant effects of warfarin.

**Methods:**

We induced TBI in mice after pre-treatment with warfarin and analyzed TBI exacerbation based on the prothrombin time-international normalized ratio (PT-INR) value, brain hemorrhage volume, blood warfarin and 7-hydroxywarfarin levels, and cytochrome P450 2C9 (CYP2C9) protein expression. C57BL/6J mice fed with a vitamin K-deficient diet received oral warfarin (low dose, 0.35 mg/kg/24 h; high dose, 0.70 mg/kg/24 h), and focal brain damage was induced in the cerebral cortices using a brain contusion device. Warfarin-treated injured mice were compared with sham-treated mice (scalp incision alone or scalp incision + bone window formation).

**Results:**

When warfarin was administered, the PT-INR value and brain hemorrhage volume associated with cerebral contusion increased on the first day post-injury. High blood warfarin and 7-hydroxywarfarin levels were observed. However, no significant differences in CYP2C9 expression were observed between the groups.

**Discussion:**

Elevated warfarin levels post-injury can increase cerebral hemorrhage risk, possibly worsening TBI. TBI might also elevate warfarin levels, heightening its anticoagulant effects. Therefore, assessing injury severity levels and PT-INR values in patients with TBI on warfarin is crucial to anticipate delayed bleeding risks.

## Introduction

Mild traumatic brain injury (TBI) is common in older adults [1]. Its incidence in people aged >60 years is globally projected to increase from 12% to 22% between 2015 and 2050. Consequently, the rates of lifestyle-related diseases and geriatric syndromes have increased [2]. TBI-related deaths associated with simple falls are correlated with current medication usage or previous falls [3]. Psychotropic drugs and some heart medications may cause sedation, balance loss, and orthostatic hypotension, contributing to falls. These are termed fall-risk-increasing drugs [4]. Furthermore, TBI may be more severe in people with pre-existing conditions than in healthy individuals.

In 2019, the World Health Organization indicated that ischemic heart disease and stroke were the top two global causes of death [5]. Patients use antithrombotic drugs to prevent blood clot formation, with warfarin commonly prescribed, particularly for atrial fibrillation [6]. Direct oral anticoagulants provide more choices, thereby decreasing warfarin usage [6]. However, many patients still take warfarin because of its cost-effectiveness and uncertainty about alternative medications [7, 8].

Older adult warfarin users with TBI have higher mortality rates and risk of worsening TBI than non-users [9, 10]. Increased TBI mortality is associated with anticoagulant (warfarin) use and age [11]. Despite the need to individualize warfarin dosage based on the prothrombin time-international normalized ratio (PT-INR) value to achieve the therapeutic goal [12], cases of inappropriate warfarin administration where the PT-INR value is not considered have been reported [13]. Therefore, administering optimal anticoagulant therapy with particular attention to high PT-INR values is essential. Additionally, careful consideration of warfarin usage is warranted in cases of TBI [14, 15].

An experimental mouse model for TBI and warfarin therapy has been established [16, 17]. Warfarin was administered to mice for 24 h to achieve therapeutic levels comparable to those in humans; these mice exhibited increased hematomas compared to warfarin-naive controls when intracerebral hemorrhage was induced [16]. Since its inception, this model has been adopted in many studies [18, 19].

However, many uncertainties regarding the impact of increased bleeding in TBI exist not only on lethality but also on long-term effects, indicating a lack of research in this field. Therefore, this study aimed to investigate the exacerbation of TBI under the influence of warfarin, comprehensively evaluate the impact of TBI on the anticoagulant effects of warfarin and gain a better understanding of TBI exacerbation. Furthermore, we aimed to contribute to the development of more effective treatment strategies and improvement of prognosis for patients with TBI undergoing warfarin therapy.

## Materials and methods

### Study design

In this study, we combined the warfarin administration model with the controlled cortical impact (CCI) model and factored in warfarin pharmacokinetics. TBI was induced in mice pre-treated with warfarin, and the mechanisms of TBI exacerbation were analyzed by measuring the PT-INR value, brain hemorrhage volume, and levels of blood warfarin and its metabolite 7-hydroxywarfarin. The commonly used warfarin is a racemic mixture of (R)-warfarin and (S)-warfarin; however, (S)-warfarin, metabolized by CYP2C9, is the most potent enantiomer. Hepatic CYP2C9 activity is a crucial determinant of inter-individual variability in anticoagulant response to warfarin [20]. Therefore, we measured the protein levels of CYP2C9 to ascertain its involvement in TBI exacerbation.

### Ethical considerations

Animal experiments were conducted in compliance with the Tokyo Women’s Medical University Animal Experimentation Regulations, following approval from the Ethical Review Committee for Animal Experiments (approval number: AE23-002).

### Chemicals and reagents

Medetomidine hydrochloride was purchased from ZENOAQ (Fukushima, Japan). Midazolam was purchased from Fuji Pharmacia (Toyama, Japan). Butorphanol was purchased from Meiji Seika Pharma (Tokyo, Japan). Warfarin sodium was purchased from FUJIFILM Wako Pure Chemical Corporation (Osaka, Japan). Finally, 7-hydroxywarfarin and warfarin-d5 were purchased from Toronto Research Chemicals, Inc. (Toronto, Canada).

### Animals, warfarin administration, and surgery

In this study, 176 male C57BL/6 mice (8–10 weeks old; CLEA, Tokyo, Japan) were assigned to the following three groups: untreated (control), pre-treated with a low warfarin dose (L-W), and pre-treated with a high warfarin dose (H-W). This count excludes three MRI-imaged mice but includes two mice that were later excluded. After 24 h of pre-treatment with warfarin, TBI was induced by CCI (TBI), or sham procedures consisting of scalp incision (SI) or SI + bone window (BW) formation were performed. Samples or parameters were collected or compared at post-injury hours (PIHs) 2, 24, and 72. The group size was calculated based on INR data from previous studies involving warfarin-treated and control mice [16], which are similar to the H-W and control groups in this study. Using G*Power v3.1.9.7, we determined an effect size of 1.23. With α error probability of 0.05 and power (1–β error probability) of 0.8, the required group size was calculated to be four mice. However, since this study includes an additional L-W and a two-factor design involving injury, we adjusted the group sizes as follows: 7–8 mice for the injury groups and at least 5 mice for the non-injury groups, with random assignment. Table 1 presents the actual group size (n) for each group, corresponding to the specified samples or parameters. However, only CYP2C9 protein expression experiments were performed using five mice. All mice were fed a no-phylloquinone diet *ad libitum* from 1 week before to the end of the experiment to eliminate the effects of vitamin K; this is a modified standard AIN-93G diet purchased from Research Diets Inc. (New Brunswick, NJ, USA). The mice were maintained on a 12-h light/dark cycle at 23 ± 1°C.

**Table 1:** Group size (n) for each group at each PIH.

	Control			L-W			H-W		
	SI	BW	TBI	SI	BW	TBI	SI	BW	TBI
PIH 2	7	5	8	5	5	8	6	5	8
PIH 24	5	5	8	6	7	7	6	5	8
PIH 72	5	7	8	6	8	8	5	5	8

BW, bone window; H-W, high warfarin dose; L-W, low warfarin dose; PIHs, post-injury hours; SI, scalp incision; TBI, traumatic brain injury

The mice received oral racemic warfarin sodium dissolved in drinking water via a water bottle for 24 h. Treatment included control, low-dose (2.33 μg/mL, 0.35 mg/kg), and high-dose (4.67 μg/mL, 0.7 mg/kg) concentrations, based on the water intake (15 mL/100 g body weight) over 24 h. We modified the warfarin dosage to approximately one-third of the dosage reported with standard mouse feed [16].

After 24 h of warfarin administration, the mice were anesthetized using a subcutaneous injection of three types of mixed anesthetic agents as follows: domitol (medetomidine hydrochloride; 0.3 mg/kg), midazolam (4 mg/kg), and butorphanol (5 mg/kg) [21]. The mice’s rectal temperature was maintained at 37°C using a warming pad with a feedback probe (Bio Research Center Co., Ltd., Aichi, Japan). After confirming that the anesthesia was effective, the mice were placed in a stereotaxic frame, and their scalps were shaved and cut open to expose the skull.

A dental trephine drill was used to create a 5-mm diameter opening, centered 3.5 mm posterior to the coronal suture and 3.5 mm lateral to the sagittal suture over the left parietal cortex [22, 23]. A CCI device (Impact One™ Stereotaxic instrument; Leica Microsystems, Wetzlar, Germany) was used to induce TBI in the TBI groups (CCI model). The device was set at a speed of 3.0 m/s and depth of 2 mm, with a dwell time of 0.5 s, and a 3-mm diameter rounded metal tip was attached. Following TBI induction, the bone flap was adhered to a plastic plate (6 mm diameter and 0.2 mm thickness) with cyanoacrylate adhesive (Aron Alpha high-speed EX, Toagosei, Japan) and restored to seal the craniotomy opening, and the scalp was closed with sutures. The mice were placed in a heat-controlled cage maintained at 38–40°C to maintain their body temperature for approximately 3 h until recovery from anesthesia. CCI model mice were compared with uninjured mice (SI or BW). Before conducting the experiments with the mice used in this study, magnetic resonance imaging (MRI) scans of one TBI mouse at each warfarin dosage were taken to confirm the damage. MRI was performed using ParaVision 6.0.1 on the Bruker Icon 1T MR imaging system (Bruker BioSpin Corp., Billerica, USA). During MRI, mice were sedated using triple-mixed anesthesia, similar to that in CCI surgery. Images were acquired using T2_RARE_Nav (T2-weighted RARE) with the following parameters: field of view, 10×15 mm; matrix, 100 × 150, slice gap, 0 μm; repetition time, 3039.023 m; echo time, 80 ms; rare factor, 12; refocusing angle, 180°; slice thickness, 0.5 mm; and total scan time, 45 min. All groups experienced identical anesthesia conditions.

### Sample collection and PT-INR measurement

At PIHs 2, 24, and 72, the mice were deeply anesthetized (100 mg/kg pentobarbital, intraperitoneal), and blood was collected via cardiac puncture. The PT-INR was measured in fresh blood drops using a Coaguchek XS coagulometer device (Roche Diagnostics, Basel, Switzerland) [24]. After blood release due to right atrial transection, the mice were transcardially perfused using pH 7.4 phosphate-buffered saline (Takara Bio, Tokyo, Japan). The liver and brain were removed, with the brains divided into left and right hemispheres, and stored at -80°C until use.

### Measurement of brain hemorrhage volume

A partially modified Foerch method was used to measure brain hemorrhage volume [16, 17]. Briefly, the brain hemispheres were placed in 2.0 mL microfuge tubes, and 1.0 mL of phosphate-buffered saline was added. The samples were crushed in a Precellys 24-bead homogenizer (Bertin Technology, Saint-Quentin, France) at 5,000 rpm for 2 × 45 s (with a 15 s break), sonicated for 1 min, and subsequently centrifuged for 30 min at 13,000 rpm (13,400 × g) and 4°C. Next, 250 μL of the supernatant was mixed with 1,000 μL of Drabkin’s reagent (Merck, Darmstadt, Germany). The absorbance was measured at 540 nm wavelength using a spectrophotometer (Beckman Coulter Inc., CA, USA). Brain hemorrhage volume was calculated based on the standard curve derived by mixing non-injury mouse brains with 0.5, 1.0, 2.0, 4.0, 8.0, 16.0, and 32.0 μL of mouse blood.

### Measurement of blood warfarin and 7-hydroxywarfarin levels using liquid chromatography-tandem mass spectrometry

Blood samples were extracted using the Quick, Easy, Cheap, Effective, Rugged, and Safe method [25]. Warfarin-d5 was used as an internal standard at 10 μg/mL. Warfarin and 7-hydroxywarfarin levels in cardiac blood samples were analyzed using a liquid chromatography-tandem mass spectrometry (LC-MS/MS) system (Shimadzu, Kyoto, Japan).

A Shima-Pack FC-ODS (2.0 × 150 mm, 3 μm) separation column with an Analytical Guard Cartridge System (Phenomenex, Torrance, CA, USA) was used. The mobile phases were 10 mM ammonium formate + water (A) and methanol (B) at a flow rate of 0.3 mL/min. Gradient elution was performed using the following program: 0–15 min from 5% to 95% B, 15–20 min B, 20–20.01 min from 95% to 5% B, and 20.01–30 min 5% B, for a total runtime of 30 min. The column oven was maintained at 40°C, and the sample injection volume was 5 μL. Mass spectrometry was performed in the positive electrospray ionization (ESI) mode (ESI+) to produce protonated analyte molecules.

The precursor and major product ions of the analytes were monitored using multiple reaction monitoring. The ion transitions of warfarin, 7-hydroxywarfarin, and warfarin-d5 were monitored at *m*/*z* 309.15/251.05/163.05, 325.33/266.95/179.0, and 314.36/256.1/160.3, respectively.

A calibration curve was prepared using blank matrices of mouse heart blood. Stock solutions for calibration curves of warfarin sodium and 7-hydroxywarfarin in methanol were used at 0.001, 0.005, 0.01, 0.05, 0.1, and 0.25 μg/mL (0.25 μg/mL using warfarin sodium alone).

The limit of detection (LOD) and limit of quantification (LOQ) were defined as the response of the analyte at the lowest concentration being 3 and 10 times greater than that of the blank sample, respectively.

### Measurement of protein expression of CYP2C9

Microsomes were isolated from liver samples using the technique previously reported by Kalsotra et al. [26, 27]. The microsomal total protein concentration was determined using a Qubit protein assay kit (Thermo Fisher Scientific, Waltham, MA, USA). Western blot with an Abby protein capillary electrophoresis device (Protein Simple, San Jose, CA, USA) was used to determine the CYP2C9 protein expression levels. Each well was loaded with 50 μg/mL of microsomes. The primary antibody, anti-rabbit polyclonal CYP2C9 (dilution 1:50; ab4236, Abcam, Cambridge, UK), and the secondary antibody, anti-rabbit horseradish peroxidase (Protein Simple), were used. CYP2C9 protein expression was normalized to total protein using the Total Protein Detection Module (Protein Simple). This experiment used a control group without warfarin treatment; in each sample, an average of two measurements was used.

### Statistical analysis

Statistical analysis was performed using JMP Pro 16.0.0 (SAS Institute Inc., Cary, NC, USA). All data are presented as boxplots; mouse data were excluded if the PT-INR values were outliers in the box plots or Smirnov–Grubbs test [28, 29].

The three groups were compared using the Kruskal–Wallis and Steel–Dwass post-hoc tests. Statistical significance for the Kruskal–Wallis test (all displayed in the graph) was set at *p<*0.05, while that for the Steel–Dwass post-hoc test was *p<*0.1 (shown in the graph).

## Results

**Fig 1:**
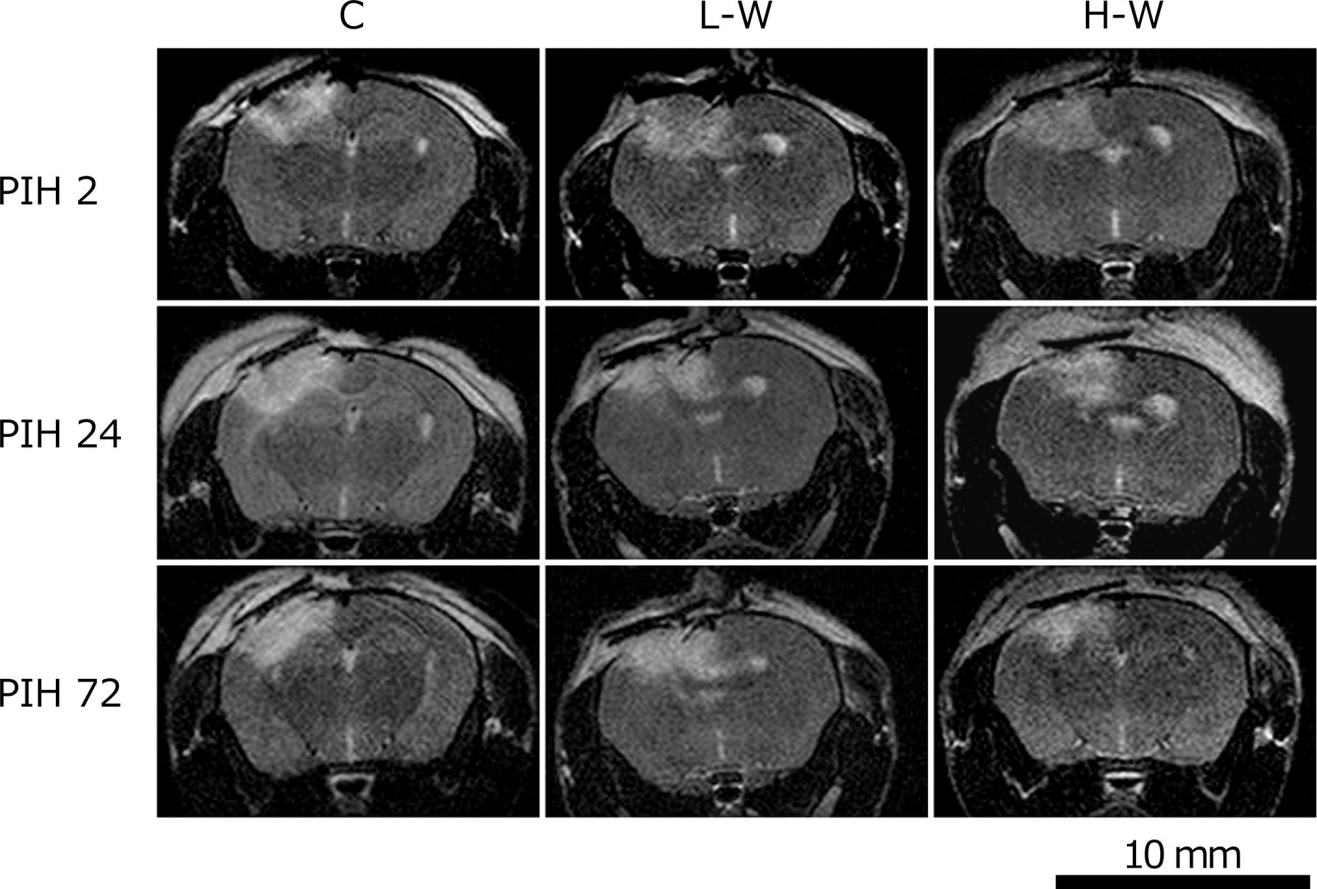
T2-weighted MRI scans for the confirmation of brain injury. One mouse from each warfarin dosage level was imaged using MRI to confirm the brain injury in TBI mice. C, control; H-W, high warfarin dose; L-W, low warfarin dose; MRI, magnetic resonance imaging; PIH, post-injury hours; TBI, traumatic brain injury.

Fig 1 shows the MRI scans taken to confirm the lesion. The MRI scans taken directly below the lesion (approximately -1.5 mm from the bregma) revealed a high-intensity signal area of approximately 10 mm^2^. No noticeable visual differences were observed in the warfarin dosage or elapsed time post-injury.

### Changes in PT-INR values

INR data for the two mice (4.2 for L-W, TBI, PIH 2; >8 for L-W, TBI, PIH 24) were identified as outliers using both boxplot analysis and the Smirnov–Grubbs test. Consequently, all data from these mice were excluded from the results.

**Fig 2:**
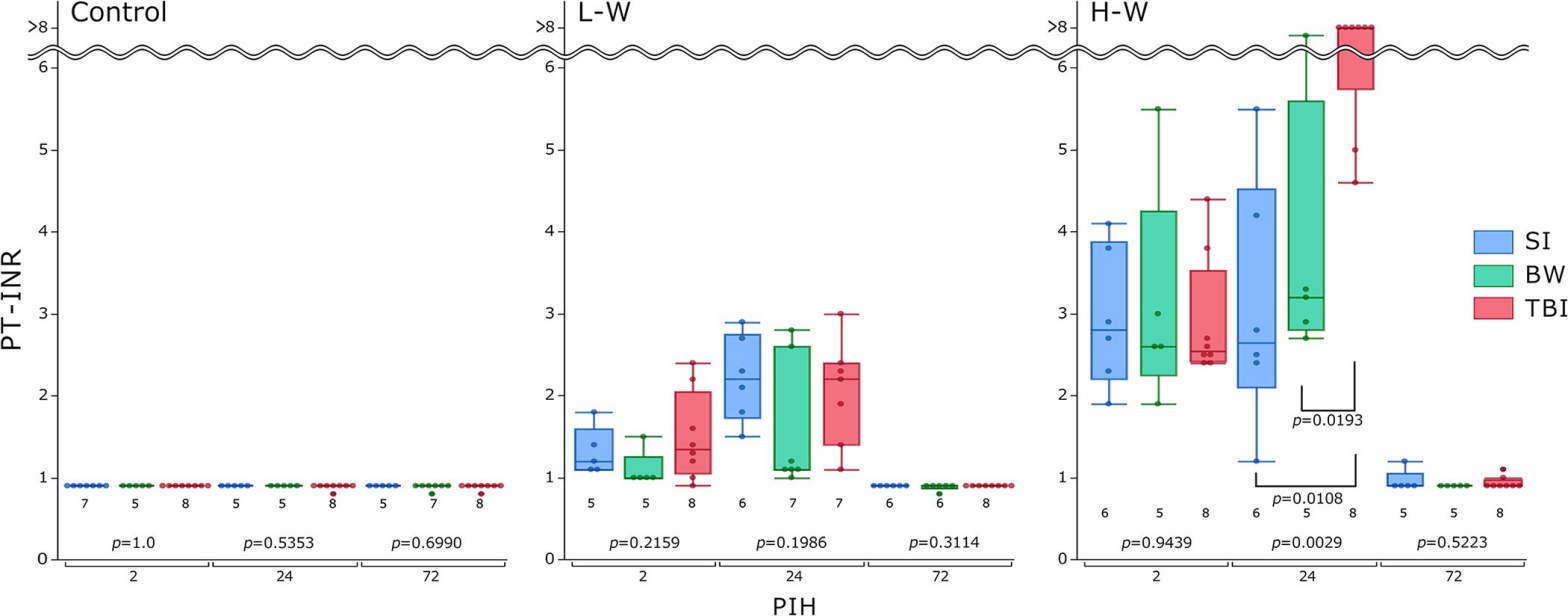
Change in PT-INR values. The results are presented as boxplots of PT-INR for the control, L-W, and H-W groups. The group size (n) for each group is indicated below the box. The *p-*values were determined using the Kruskal–Wallis test at each PIH to compare the SI, BW, and TBI groups; *p-*values <0.1, as determined using the Steel–Dwass post-hoc test, are indicated below the square brackets. BW, bone window; PIHs, post-injury hours; H-W, high warfarin dose; L-W, low warfarin dose; PT-INR, prothrombin time-international normalized ratio; SI, scalp incision; TBI, traumatic brain injury.

The PT-INR values, assessing the anticoagulant effect of warfarin (Fig 2), ranged from 0.8 to 0.9 (normal range in humans: 0.8–1.2) [30] in the control groups. No significant differences were observed in the groups at all PIHs at control and L-H. At PIH 24 among mice receiving H-W, the TBI group showed a significant increase in PT-INR compared with that in the SI or BW group (*p =* 0.0029). However, the PT-INR returned to normal at PIH 72 in most mice receiving H-W, but some exhibited values higher than normal in the SI and TBI groups. Supplementary comparisons of PT-INR by warfarin administration levels for each group at each PIH are shown in S1 Fig. At PIHs 2 and 24 in mice receiving L-W, a PT-INR value higher than normal was observed, returning to normal levels at PIH 72. At PIHs 2 and 24, the PT-INR significantly increased in the H-W groups compared to that in the L-W groups in nearly all cases. Furthermore, at PIH 72, no significant differences in warfarin administration levels were observed within any of the groups.

### Brain hemorrhage volume

**Fig 3:**
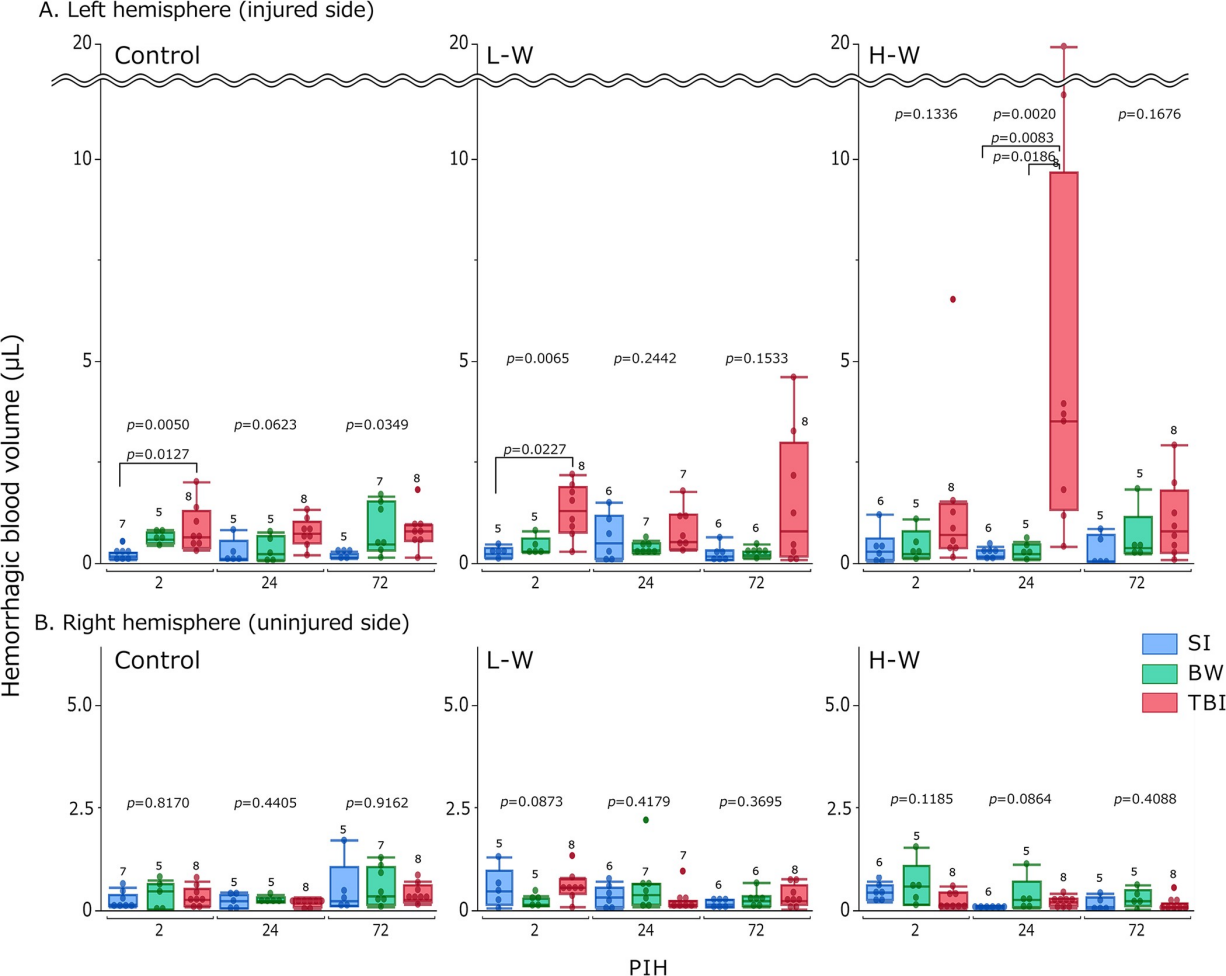
Changes in brain hemorrhage volume in each hemisphere. The results are presented as boxplots of the brain hemorrhage volume in the left (A) and right (B) hemispheres after TBI for the control, L-W, and H-W groups. The group size (n) for each group is indicated below the box. The *p-*values were determined using the Kruskal–Wallis test at each PIH to compare the SI, BW, and TBI groups; *p-*values <0.1, as determined using the Steel–Dwass post-hoc test, are indicated above the square brackets. BW, bone window; H-W, high warfarin dose; L-W, low warfarin dose; PIHs, post-injury hours; SI, scalp incision; TBI, traumatic brain injury.

This was measured in each hemisphere to evaluate the effect of warfarin on hemorrhage in TBI (Fig 3). In the left hemisphere (injured side) (Fig 3A), at PIHs 2 and 72 among the controls, a significant difference was observed between the groups (*p =* 0.0050, PIH 2 and 0.0349, PIH 72), and the TBI group tended to have increased brain hemorrhage volumes. Furthermore, no significant difference was observed among the controls at PIH 24. At L-W, significant differences at PIH 2 (*p =* 0.0065), and the TBI group tended to have increased brain hemorrhage volume. However, at L-W, no significant difference was observed between groups at PIHs 24 and 72. At H-W, a significant difference was observed between groups at PIH 24 (*p =* 0.0020); TBI significantly increased brain hemorrhage volume compared to SI (*p =* 0.0083) and BW (*p =* 0.0186). At H-W, no significant difference was observed between the groups at PIHs 2 and 72. All right hemispheres (non-injured side) showed no significant differences among all groups at all PIHs (Fig 3B). Supplementary comparisons of brain hemorrhage volume by warfarin administration levels for each group at each PIH are shown in S2 Fig. In the TBI group at PIH 24, a significant difference was observed in brain hemorrhage volume of the left hemisphere according to the warfarin administration levels (*p =* 0.0147). However, no significant differences were observed at other PIH points and in the other groups.

### Blood warfarin levels

**Fig 4:**
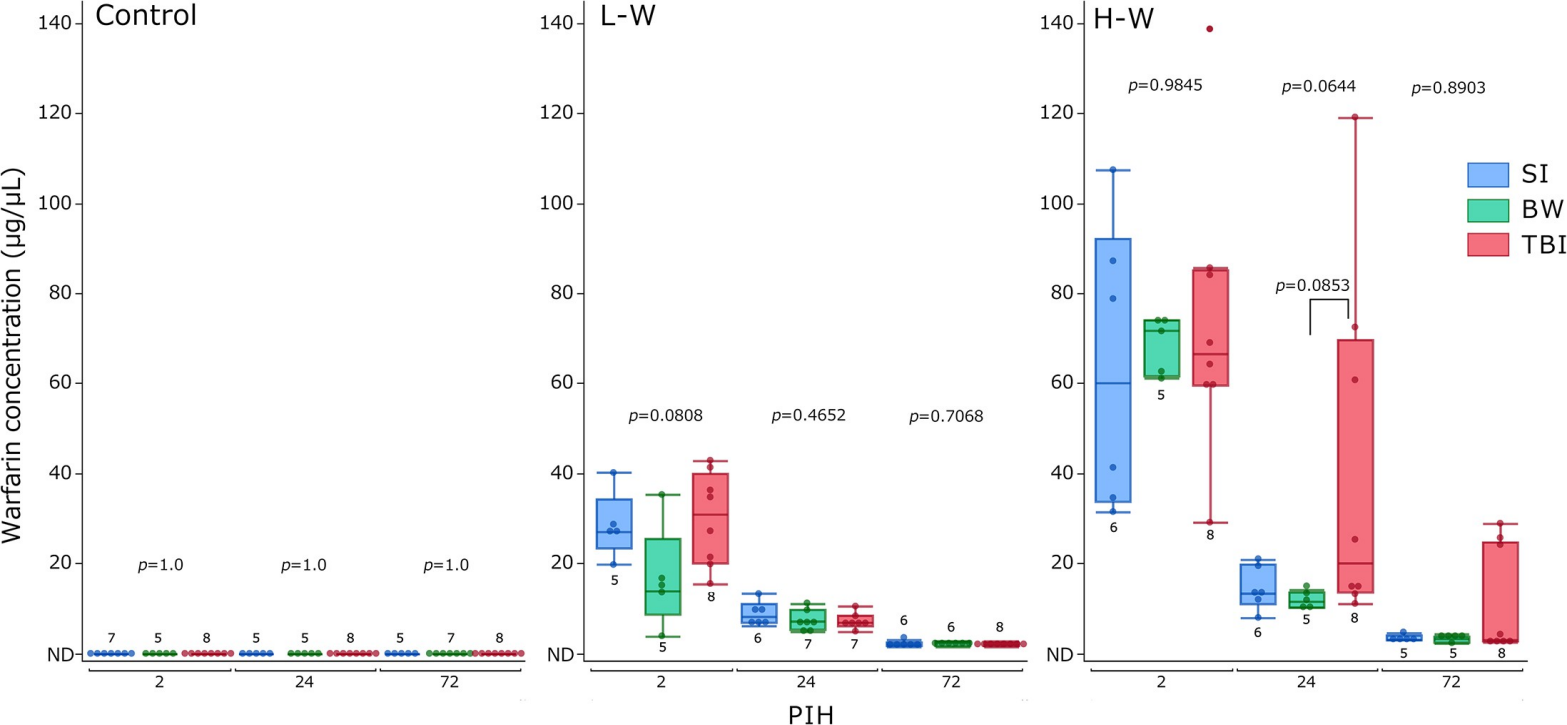
Changes in blood warfarin levels. The results are presented as boxplots of blood warfarin levels for the control, L-W, and H-W groups. The group size (n) for each group is indicated below or above the box. The *p-*values were determined using the Kruskal–Wallis test at each PIH to compare the SI, BW, and TBI groups; *p-*values <0.1, determined using the Steel–Dwass post-hoc test, are indicated above the square brackets. BW, bone window; H-W, high warfarin dose; L-W, low warfarin dose; ND, not detected; PIHs, post-injury hours; SI, scalp incision; TBI, traumatic brain injury.

Blood warfarin concentrations were measured using LC-MS/MS (Fig 4). The LOD and LOQ were 0.1 and 0.5 μg/μL, respectively. In the control groups, blood warfarin was not detected. No significant differences existed between the groups receiving L-W at any PIH. At L-W, the warfarin concentration was the highest across all groups at PIH 2 and subsequently declined. At H-W, no significant difference was observed among the groups at all PIHs. At H-W, the TBI group at PIHs 24 and 72 showed a trend toward higher blood warfarin concentrations than the non-injured groups. Supplementary comparisons of blood warfarin levels according to the warfarin administration levels for each group at each PIH are shown in S3 Fig. At all PIHs, higher warfarin administration levels resulted in significantly increased blood warfarin concentrations across all groups.

### Blood 7-hydroxywarfarin levels

**Fig 5:**
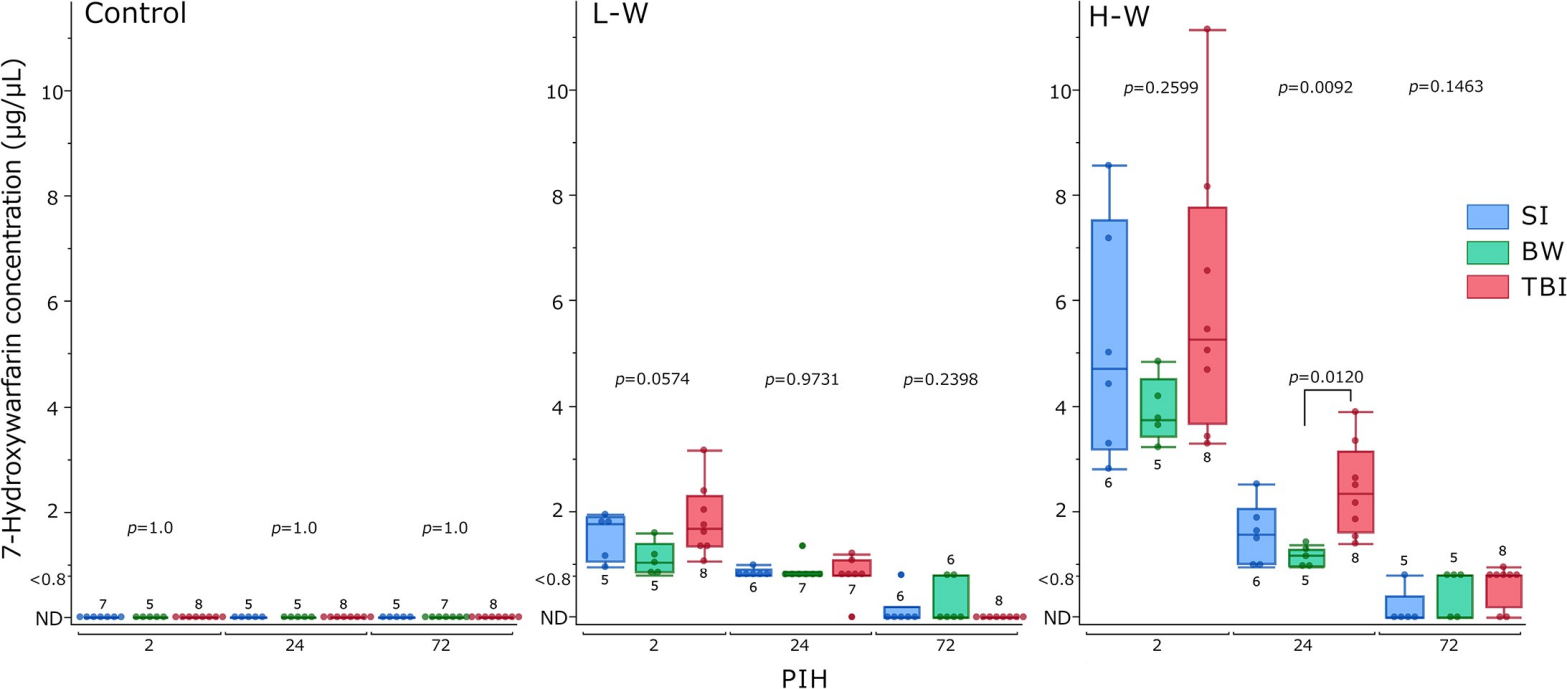
Changes in blood 7-hydroxywarfarin levels. The results are presented as boxplots of blood 7-hydroxywarfarin levels for the control, L-W, and H-W groups. The group size (n) for each group is indicated below or above the box. The *p-*values were determined using the Kruskal–Wallis test at each PIH to compare the SI, BW, and TBI groups; *p-*values <0.1, as determined using the Steel–Dwass post-hoc test, are indicated above the square brackets; BW, bone window; H-W, high warfarin dose; L-W, low warfarin dose; ND, not detected; PIHs, post-injury hours; SI, scalp incision; TBI, traumatic brain injury.

Blood 7-hydroxywarfarin levels were measured using LC-MS/MS (Fig 5). The LOD and LOQ were 0.3 and 0.8 μg/μL, respectively. In the control groups, 7-hydroxywarfarin was not detected. Furthermore, the L-W groups showed no significant differences at any PIH. The H-W showed no significant differences at PIHs 2 and 72. However, at PIH 24, the TBI group showed a significant increase (*p =* 0.0120) compared with the BW group. The TBI group tended to have higher blood 7-hydroxywarfarin levels than the BW group. Supplementary comparisons of blood 7-hydroxywarfarin levels by warfarin administration levels for each group at each PIH are shown in S3 Fig. At PIHs 2 and 24, higher warfarin administration levels resulted in significantly increased blood 7-hydroxywarfarin concentrations across all groups. At PIH 72, higher warfarin administration levels resulted in significantly increased blood 7-hydroxywarfarin concentrations in the TBI group(*p =* 0.0107), but no differences were observed in the SI and BW groups.

### CYP2C9 protein expression

**Fig 6:**
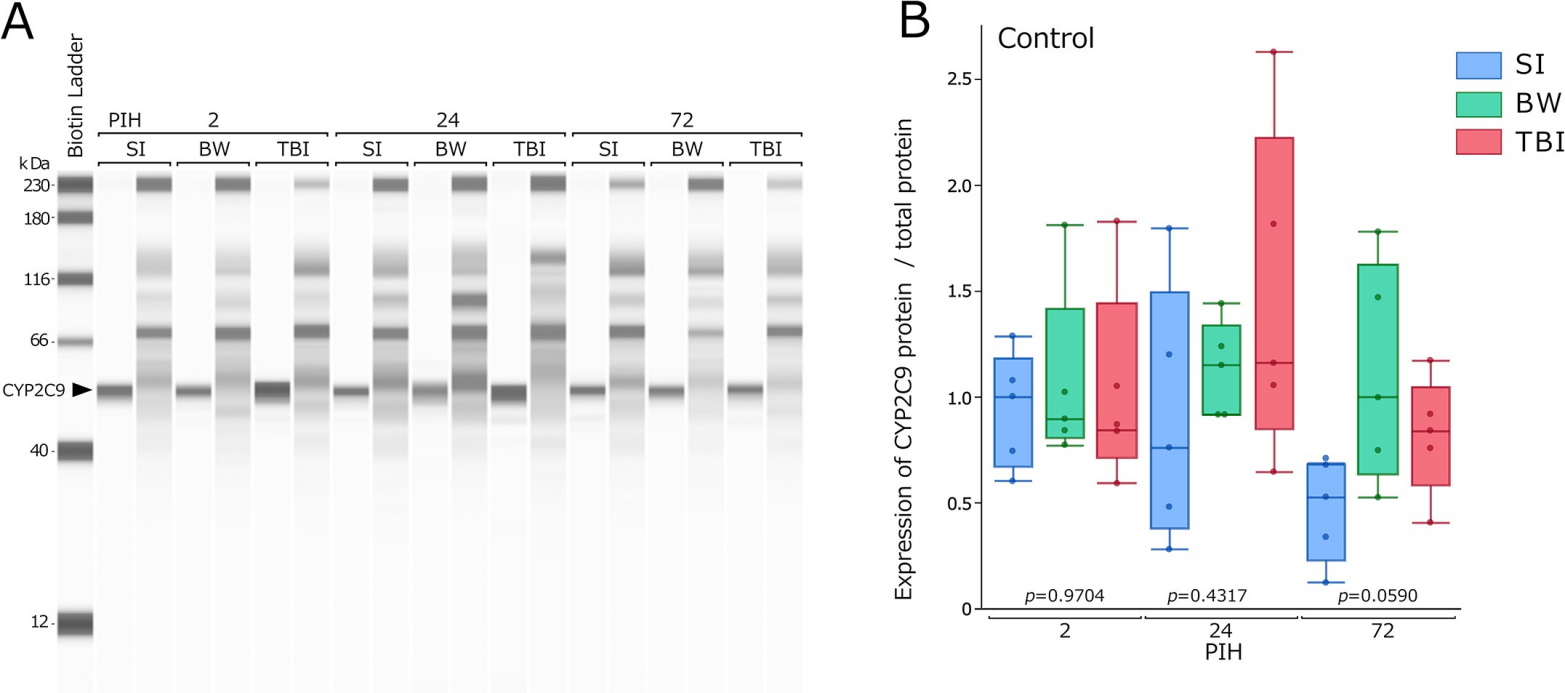
Change in CYP2C9 protein expression. (A) Western blot shows CYP2C9 expression; CYP2C9 positive signals (left) and total proteins (light) obtained from capillary protein electrophoresis are shown. (B) The results are presented as boxplots of CYP2C9 protein levels, measured using western blot, for normalization by the total protein level. All CYP2C9 protein expression results were obtained using control group mice (all groups n = 5). The *p-*values were determined using the Kruskal–Wallis test at each PIH to compare the SI, BW, and TBI groups. BW, bone window; PIHs, post-injury hours; SI, scalp incision; TBI, traumatic brain injury.

Fig 6A depicts a representative full-length western blot of CYP2C9, and Fig 6B shows the corresponding expression levels. CYP2C9 expression showed no significant differences between the groups at any PIH.

## Discussion

In this study, warfarin treatment increased the PT-INR value and cerebral contusion-related brain hemorrhage volume on the first day post-injury. An important finding of this study is that, in the TBI group, an increase in blood warfarin and blood 7-hydroxywarfarin levels were noted on PIH 24 compared with the non-injured group. No significant differences were observed in CYP2C9 protein expression between the groups.

In neuroscience, female mice have historically been avoided to circumvent the influence of hormonal cycles; however, in 2014, the National Institutes of Health recommended the inclusion of both sexes. Although estrous cycles and sex differences raise concerns about data variability, some studies suggest that it may not be necessary to account for these cycles [31–33]. On the other hand, the influence of sex hormones on inflammatory responses during injury is established [34], suggesting that the use of both male and female mice warrants further consideration. In this study, only male mice were used, as the purpose was not to investigate sex differences. This decision also follows prior research on warfarin administration with CCI surgery [17] and prioritizes minimizing animal use in line with the 3Rs principles (Replacement, Reduction, and Refinement) [35, 36].

Here, we adopted the warfarin administration method based on the literature [16] but adjusted the concentration to account for the impact of using vitamin K-deficient feed. Specifically, we set the warfarin concentration at approximately one-third (0.70 mg/kg/24 h, H-W) of that used in the previous study (2.0 mg/kg/24 h). Consequently, PT-INR values similar to those in the literature were obtained 2 h later (range 2.0–4.5 in the literature [16]; 1.9–4.1 in this study). The American College of Chest Physicians guidelines indicate that the therapeutic range of PT-INR is 2.0–3.0 [12]. These values can be considered to be within the expected clinical range.

In this study, an increase in brain hemorrhage volume occurred in the TBI group at PIH 24 on H-W. The brain hemorrhage volume in the warfarin-treated mice undergoing CCI surgery in a previous study [30] was comparable to our results. However, the occurrence of delayed intracranial hemorrhage in patients with TBI on anticoagulant therapy is still debated. Some patients may face delayed bleeding with significant consequences. Patients with PT-INR value of >2 or >3 post-TBI should be closely monitored, with regular head computed tomography recommended [37, 38]. Our results indicated increased brain hemorrhage volume in the TBI group at PIH 24, likely linked to the previously mentioned delayed bleeding.

At PIH 24, the TBI group on H-W had significantly higher blood warfarin levels in three of eight samples than the non-injured groups. At PIH 72, the SI and BW groups on H-W had decreased blood warfarin levels, while the TBI group maintained the levels in three of the eight samples. This implies that patients with TBI on H-W have higher blood warfarin levels than those without TBI. Specifically, it implies that TBI elevates the blood warfarin levels. In essence, this suggests that warfarin does not only worsen TBI, but TBI also increases the effects of warfarin through increased blood concentration, leading to further deterioration of TBI. Our results provide new challenges and warnings in the combination of pharmacokinetics and traumatology.

The TBI group receiving H-W tended to have elevated 7-hydroxywarfarin levels at PIH 24. CYP2C9 in cytochrome P450 (CYP450) metabolizes warfarin to 7-hydroxywarfarin [20]. CYP450 expression varies with organ, sex, and genetic mutations [18, 26]. A previous study using a rat CCI model found that hepatic CYP1A levels decreased in the injured group 24 h and 2 weeks post-injury, while the levels of other CYP450 enzymes remained stable or increased [26]. CYP450 expression is believed to be initially suppressed by cytokine upregulation due to damage, followed by an increase through protein supplementation [27, 39, 40]. Our study results did not indicate reduced CYP2C9 or 7-hydroxywarfarin levels post-TBI, but a slight upward trend was observed at PIH 24. CYP2C9 is induced by interleukin-6, which is reported to peak 6 h post-TBI in the rat CCI model [41, 42]. Therefore, the decrease in CYP2C9 and 7-hydroxywarfarin levels might not have been detected at the time points of our experiments.

In this study, a significant increase in PT-INR, brain hemorrhage volume, and blood warfarin levels at PIH 24 was observed in the TBI group on H-W compared to the values in the SI and BW groups, but not in the TBI group on L-W. The dose-response relationship, indicating the association between the dose of a drug and its response, demonstrates a sigmoid curve. In the curve, a linear proportional relationship exists within the therapeutic window, spanning from effective to toxic doses. Effects weaken outside this dose range [43]. Warfarin has a steep dose-response curve, indicating a narrow therapeutic window, resulting in a reduced response when the dose is outside the range [44]. In this study, no difference was observed between the TBI and non-injured groups on L-W treatment, and this may be attributed to the L-W dosage falling below the therapeutic range, resulting in a blunted response. Conversely, the difference observed between the TBI and non-injured group on H-W treatment is likely because the H-W enters the therapeutic window range in the warfarin dose response, resulting in heightened sensitivity.

To mitigate the severity of injuries in patients with TBI taking warfarin, predicting experimentally suggested delayed bleeding by experiments and accordingly devising the most effective measures is imperative. With H-W at PIH24, where the brain hemorrhage volume increased post-TBI, PT-INR values and blood warfarin and 7-hydroxywarfarin levels also increased. Therefore, measuring their blood levels is likely to aid in predicting the extent of a brain hemorrhage. In postmortem examinations where obtaining fresh blood is particularly challenging, the PT-INR value cannot be measured. However, by understanding the intake amount and timing of warfarin and subsequently measuring its blood concentrations, assessing the extent of increased brain hemorrhage volume may be possible, ultimately leading to the accurate determination of the cause of death. The most effective approach to reduce the severity of emergency patients is to promptly reverse warfarin effects post-injury, thereby minimizing bleeding duration and reducing blood loss. In clinical settings, vitamin K or fresh frozen plasma is preferred to rapidly reverse warfarin-induced coagulopathy, and prothrombin complex concentrate (PCC) is usually used in emergencies [39–47]. In an animal study, mice treated with warfarin and administered PCC 60 min after CCI exhibited a significantly lower brain hemorrhage volume than those given saline [28]. In emergency medical services, administering PCC to patients with TBI on warfarin is recommended if the injury becomes life-threatening. However, evidence suggests that it does not effectively reduce mortality and is associated with an increased risk of thrombotic events [48, 49]. Based on the results of this study, implementing safer and more effective strategies for mitigating injury exacerbation by accurately understanding the risk of worsening damage before using these medications should be possible, thereby minimizing the risk of thrombosis. For future investigations, conducting experiments, including animal studies, to explore the timing of drug administration and warfarin discontinuation/resumption is considered effective.

In this study, behavioral investigations were discontinued. The research time points were set in accordance with the metabolism speed of warfarin up to PIH 72. Considering previous research [50], the period for behavioral evaluation was insufficient in our study. Furthermore, this study involved a dual investigation of warfarin administration and TBI in a mouse model. Adding behavioral tests to mice should be undertaken only when essential for the experiment because it may introduce undesirable variations in the results [51, 52]. Nevertheless, reports on behavioral experiments in mice with TBI administered with warfarin are limited: a study reported a peak in functional impairment on day 3 post-injury in the warfarin-treated group using the wire-grip test [53]; however, neither Schaefer et al. [53] nor Foerch et al. [17] observed significant functional decline during the subsequent month. Moreover, none of the studies found an influence of warfarin administration on the Neurological Severity Score [53] and Morris water maze test result [17]. Given the dissociation observed between histopathology (lesion size) and functional recovery in several TBI models [53, 54], evaluating deterioration using various analyses, not solely relying on the results of behavioral experiments, is necessary.

Several important studies [16, 17, 39], including this study, were conducted using animal models. Therefore, directly applying the findings of these studies to humans might be challenging. This aspect is considered a limitation of these studies. To address this, recognizing the discrepancies in research outcomes between animals and humans is crucial. As one potential solution, understanding the pharmacokinetics in both humans and animals and accurately comprehending the differences in their anticoagulant responses can be considered. In this study, human samples were not used; however, the normal range of PT-INR is common between mice and humans (approximately 0.9 by non-injury mice) [16, 30], and the results can be extrapolated to humans to some extent.

## Conclusion

Our study suggests that administering H-W increases PT-INR value and the risk of brain hemorrhage associated with a cerebral contusion on the first day post-injury. Moreover, high blood warfarin and 7-hydroxywarfarin levels were observed. No significant differences in CYP2C9 expression were observed between the groups. The elevated blood levels of warfarin observed in our results on the day post-injury are believed to increase the risk of brain hemorrhage and contribute to the onset of delayed bleeding. This suggests involvement in TBI exacerbation. Furthermore, this study revealed the potential for TBI to elevate blood warfarin levels, which may increase the anticoagulant effects of the drug. This suggests that TBI and warfarin mutually exacerbate each other, leading to adverse outcomes. Therefore, in diagnosing and treating patients with TBI on warfarin, assessing the severity of brain injury and their PT-INR value and anticipating potential delayed bleeding risks are crucial.

## Supporting information

S1 Fig Change in PT-INR values based on warfarin dose. The results are presented as boxplots of PT-INR categorized by PIH and groups. The group size (n) for each group is indicated below the box. The *p-*values were determined using the Kruskal–Wallis test at each PIH by group to compare the control, L-W, and H-W groups; *p-*values <0.1, as determined using the Steel–Dwass post-hoc test, are indicated above the square brackets. BW, bone window; C, control; H-W, high warfarin dose; L-W, low warfarin dose; PIH, post-injury hours; PT-INR, prothrombin time-international normalized ratio; SI, scalp incision; TBI, traumatic brain injury.
(TIF)

S2 Fig Change in brain hemorrhage volume based on warfarin dose. The results are presented as boxplots of brain hemorrhage volume categorized by PIH and groups. The group size (n) for each group is indicated above the box. The *p-*values were determined using the Kruskal–Wallis test at each PIH by group to compare the control, L-W, and H-W groups; *p-*values <0.1, as determined using the Steel–Dwass post-hoc test, are indicated above the square brackets. BW, bone window; C, control; H-W, high warfarin dose; L-W, low warfarin dose; PIH, post-injury hours; SI, scalp incision; TBI, traumatic brain injury.
(TIF)

S3 Fig Change in blood warfarin level based on warfarin dose. The results are presented as boxplots of blood warfarin levels categorized by PIH and groups. The group size (n) for each group is indicated above the box. The *p-*values were determined using the Kruskal–Wallis test at each PIH by group to compare the control, L-W, and H-W groups; *p-*values <0.1, as determined using the Steel–Dwass post-hoc test, are indicated above the square brackets. BW, bone window; C, control; H-W, high warfarin dose; L-W, low warfarin dose; PIH, post-injury hours; SI, scalp incision; TBI, traumatic brain injury.
(TIF)

S4 Fig Change in blood 7-hydroxywarfarin level based on warfarin dose. The results are presented as boxplots of blood 7-hydroxywarfarin levels categorized by PIH and groups. The group size (n) for each group is indicated above the box. The *p-*values were determined using the Kruskal–Wallis test at each PIH by group to compare the control, L-W, and H-W groups; *p-*values <0.1, as determined using the Steel–Dwass post-hoc test, are indicated above the square brackets. BW, bone window; C, control; H-W, high warfarin dose; L-W, low warfarin dose; PIH, post-injury hours; SI, scalp incision; TBI, traumatic brain injury.
(TIF)
