# Supplementary figures and images for: Mechanism of exacerbation of traumatic brain injury under warfarin anticoagulation in male mice
Source: PLoS One. 2024 Dec 5;19(12):e0314765. doi: 10.1371/journal.pone.0314765 (PMC11620684; doi:10.1371/journal.pone.0314765)

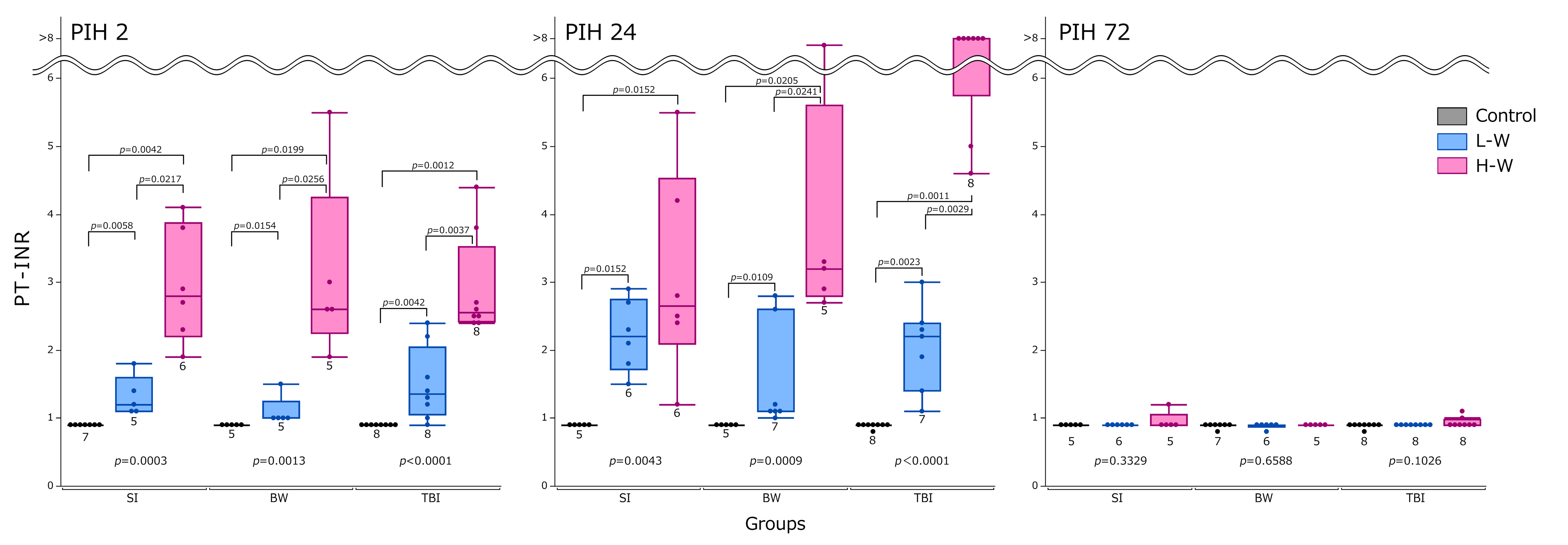

Supplement: S1 Fig — The results are presented as boxplots of PT-INR categorized by PIH and groups. The group size (n) for each group is indicated below the box. The p-values were determined using the Kruskal–Wallis test at each PIH by group to compare the control, L-W, and H-W groups; p-values <0.1, as determined using the Steel–Dwass post-hoc test, are indicated above the square brackets. BW, bone window; C, control; H-W, high warfarin dose; L-W, low warfarin dose; PIH, post-injury hours; PT-INR, prothrombin time-international normalized ratio; SI, scalp incision; TBI, traumatic brain injury. (TIF) [file pone.0314765.s001.tif]

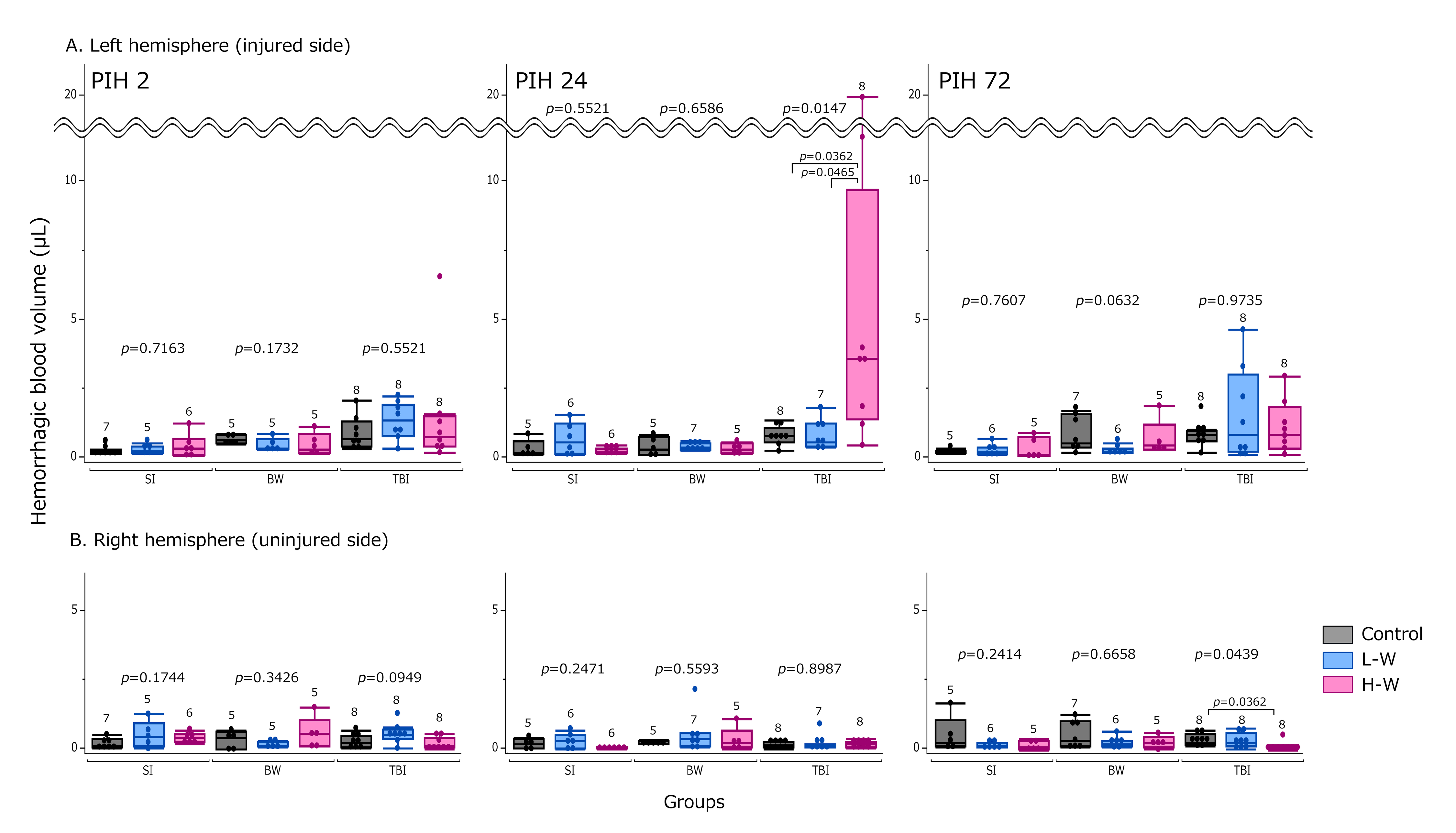

Supplement: S2 Fig — The results are presented as boxplots of brain hemorrhage volume categorized by PIH and groups. The group size (n) for each group is indicated above the box. The p-values were determined using the Kruskal–Wallis test at each PIH by group to compare the control, L-W, and H-W groups; p-values <0.1, as determined using the Steel–Dwass post-hoc test, are indicated above the square brackets. BW, bone window; C, control; H-W, high warfarin dose; L-W, low warfarin dose; PIH, post-injury hours; SI, scalp incision; TBI, traumatic brain injury. (TIF) [file pone.0314765.s002.tif]

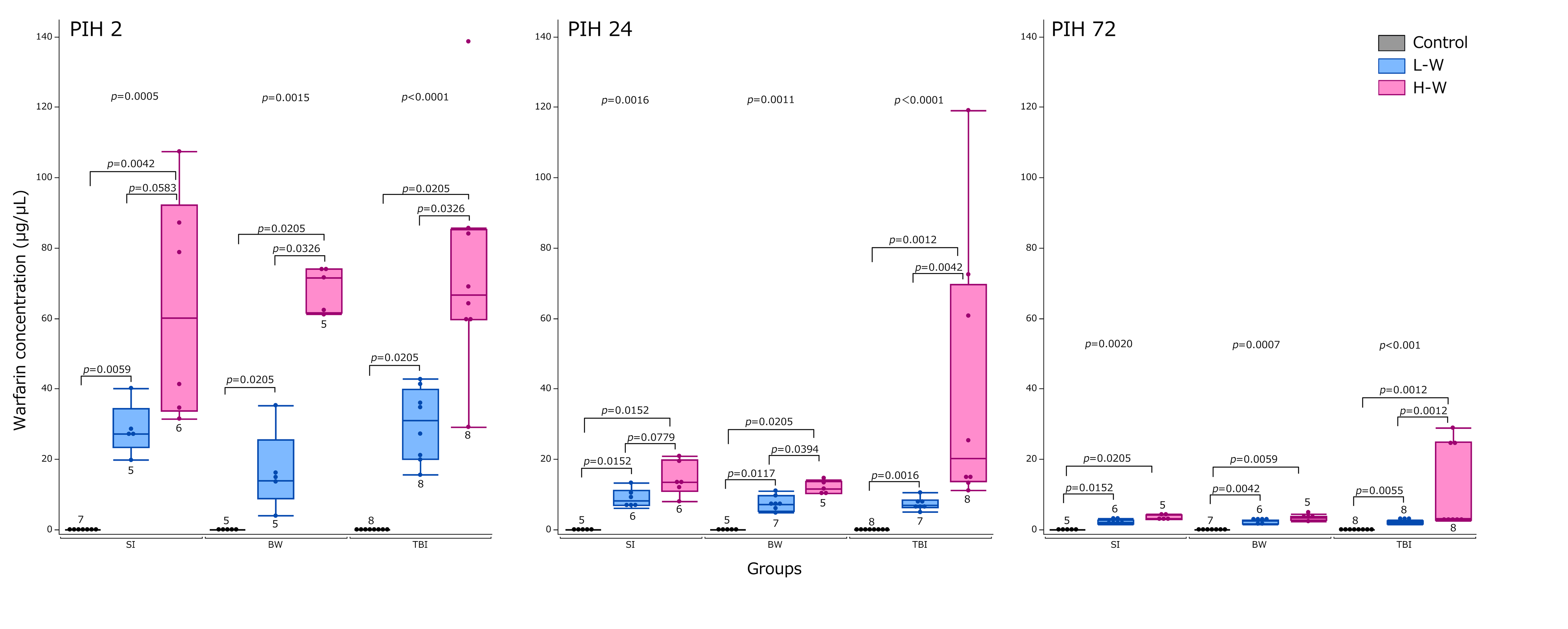

Supplement: S3 Fig — The results are presented as boxplots of blood warfarin levels categorized by PIH and groups. The group size (n) for each group is indicated above the box. The p-values were determined using the Kruskal–Wallis test at each PIH by group to compare the control, L-W, and H-W groups; p-values <0.1, as determined using the Steel–Dwass post-hoc test, are indicated above the square brackets. BW, bone window; C, control; H-W, high warfarin dose; L-W, low warfarin dose; PIH, post-injury hours; SI, scalp incision; TBI, traumatic brain injury. (TIF) [file pone.0314765.s003.tif]

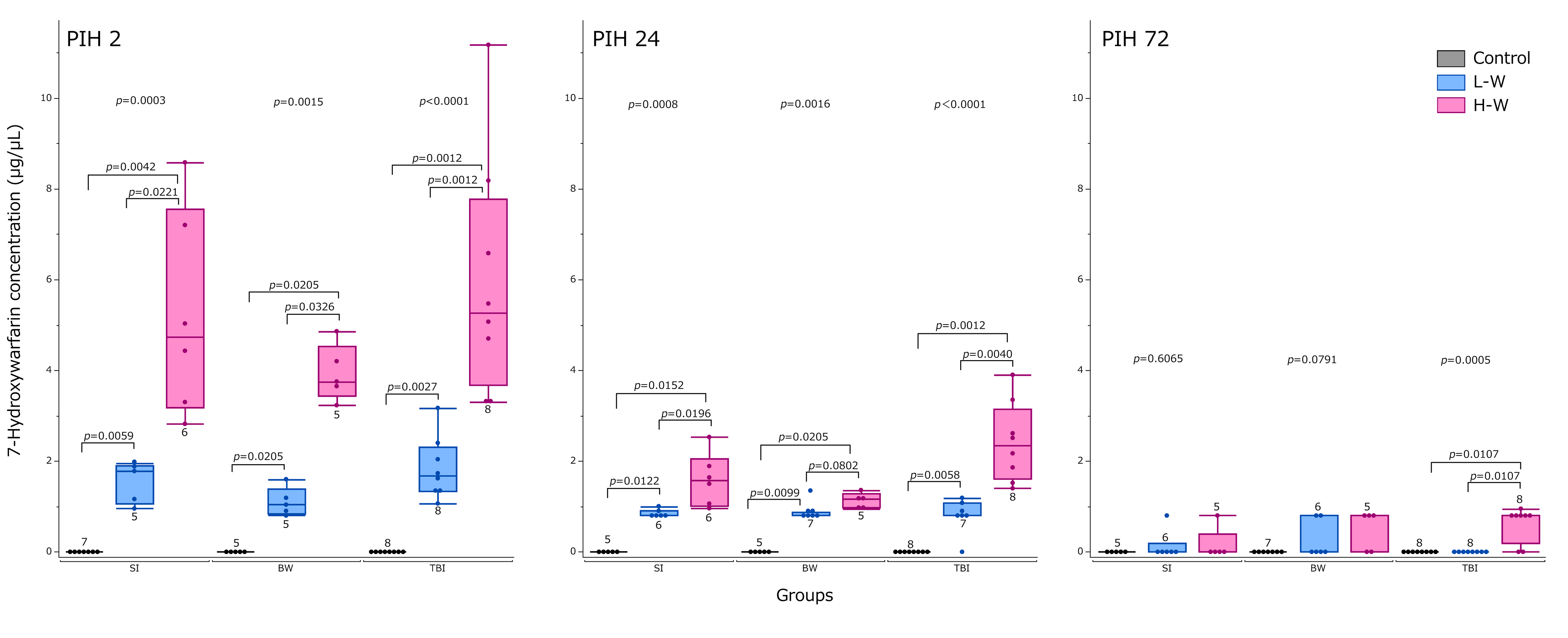

Supplement: S4 Fig — The results are presented as boxplots of blood 7-hydroxywarfarin levels categorized by PIH and groups. The group size (n) for each group is indicated above the box. The p-values were determined using the Kruskal–Wallis test at each PIH by group to compare the control, L-W, and H-W groups; p-values <0.1, as determined using the Steel–Dwass post-hoc test, are indicated above the square brackets. BW, bone window; C, control; H-W, high warfarin dose; L-W, low warfarin dose; PIH, post-injury hours; SI, scalp incision; TBI, traumatic brain injury. (TIF) [file pone.0314765.s004.tif]
